# National Immunization Program Information System: implementation context assessment

**DOI:** 10.1186/s12913-020-05175-9

**Published:** 2020-04-21

**Authors:** Brener Santos Silva, Eliete Albano de Azevedo Guimarães, Valéria Conceição de Oliveira, Ricardo Bezerra Cavalcante, Marta Macedo Kerr Pinheiro, Tarcísio Laerte Gontijo, Samuel Barroso Rodrigues, Ana Paula Ferreira, Humberto Ferreira de Oliveira Quites, Ione Carvalho Pinto

**Affiliations:** 1 PhD student in Public Health Nursing Program at the Ribeirão Preto College of Nursing at the University of São Paulo (EERP-USP), Ribeirão Preto (SP), Brazil; 2 Doctor in Health Sciences, Adjunct Teacher of the Nursing Course, Federal University of São João del-Rei (UFSJ), Campus Centro-Oeste, Divinópolis (MG), Brazil; 3 Doctor in Health Sciences, Ribeirão Preto College of Nursing at the University of São Paulo (EERP-USP), Adjunct Teacher of Federal University of São João del-Rei (UFSJ), Campus Centro-Oeste, Divinópolis (MG), Brazil; 4 Doctor in Information Science from the Federal University of Minas Gerais (UFMG), Associate Teacher of the Nursing Course, Federal University of Juiz de Fora (UFJF), Campus Juiz de Fora , Juiz de Fora (MG), Brazil; 5 Doctor in Information Science (Eco / IBICT-UFRJ), Collaborating Teacher and Researcher of the PPG-GOC Program of the Federal University of Minas Gerais, Teacher and Researcher of Fumec University, Belo Horizonte, Brazil; 6 Doctor in Health Sciences, Child and Adolescent Health, UFMG Medical School, Adjunct Teacher of Federal University of São João Del Rei (UFSJ), Campus Centro-Oeste, Divinópolis (MG), Brazil; 7 Pos-doctorate in Collective Health at the Federal University of São João del Rei (UFSJ), Campus Centro-Oeste, Divinópolis (MG), Brazil; 8 Graduating of the Nursing Course, Federal University of São João del-Rei (UFSJ), Campus Centro-Oeste, Divinópolis (MG), Brazil; 9 Doctor in Nursing, Federal University of Minas Gerais (UFMG), Adjunct Teacher of the Federal University of São João Del Rei (UFSJ), Campus Centro-Oeste, Divinópolis (MG), Brazil; 10 Doctor in Nursing from the University of São Paulo, Associate Teacher III at the Ribeirão Preto College of Nursing at the University of São Paulo (EERP-USP), Ribeirão Preto (SP), Brazil

**Keywords:** Immunization, Immunization programs, Information systems, Public health nursing, Health assessment

## Abstract

**Background:**

The National Immunization Program Information System (SIPNI - *Sistema de Informação do Programa Nacional de Imunização*) in Brazil is a technological innovation management tool that enhances the performance of managers and health professionals in the evaluation and monitoring of immunization activities. In the country, the decentralization of the System is at an advanced stage, but it still faces challenges regarding its operation and use, impacting on its results. This study aims to evaluate the deployment of SIPNI in the state of Minas Gerais, in 2017.

**Method:**

Cross-section study performed in Primary Healthcare vaccination rooms in 54 municipalities in the Brazilian state of Minas Gerais, in 2017. A multidimensional questionnaire was used with nursing professionals who work in vaccination rooms, containing questions about the structure (presence of an internet-connected computer, instruction manual, software version, IT professional for technical support, trained healthcare professional, use of communication channels to obtain system information) and the process (activities performed by the staff to operate the immunization information system) of their work. Those questions refer to the components of the information system: system management, immunized-patient records, and Movement of Immunobiological. Implementation Degree (ID) was defined by a score system with different weights for each criterion, according to the importance level observed in it, with a rating of: adequate, partially adequate, inadequate and critically inadequate. For data analysis, median was used as the summary measure, and Pearson’s Chi-Squared Test was used for proportion comparison.

**Results:**

Municipal SIPNI is not adequately implemented and that results mainly from the actions performed in health service units, indicating problems in the use of technology by professionals working in vaccination rooms. The structure was better evaluated than the process, presenting IDs of 70.9 and 59.5%, respectively. Insufficient internet access, inadequate use of communication channels, and lack of professional qualification were some of the identified structural issues. “Movement of Immunobiological” was the best-ranked component (ID = 68.5%), followed by “immunized patient records” (ID = 59.3%) and “SIPNI management” (ID = 50.7%). Partial performance of SIPNI is independent of population size in the municipality and of FSH coverage.

**Conclusions:**

SIPNI is still an underutilized technological innovation. There are challenges that must be overcome, such as implementation of the final web version, internet connectivity, and capabilities aimed at the use of information generated by technology. Nevertheless, perspectives regarding SIPNI are positive, with functionalities to optimize activities in vaccination rooms.

## Background

Acknowledgement of the importance of information in decision-making processes has promoted the development and implementation of several Health Information Systems (HIS) [1]. These systems comprise strategies of technological innovation that expedite data collection, processing and analysis, and dissemination of knowledge, enhancing information management in the various fields of health care [2, 3].

Among existing information systems, those that are able to prioritize and manage data related to immunization are especially noteworthy. Immunization Information Systems (IIS) used since the 1970s in the United States of America (USA) seek to support services in the planning and decision-making of vaccination activities [4, 5]. These are considered confidential, population-based devices whose purpose is to group, keep, and consolidate information to subsidize immunization actions at different levels of health care, especially at local levels [4].

In Brazil, the National Immunization Program (NIP), in partnership with the Brazilian Health Informatics Department (DATASUS - *Departamento de Informática do Sistema Único de Saúde*), implemented in 2010 the National Immunization Program Information System (SIPNI - *Sistema de Informação do Programa Nacional de Imunização*). This system incorporates, on a single base, subsystems that provide information on individual dosage records, vaccine coverage, immunobiological agent inventory control, and indications for special immunobiological agents and post-vaccination adverse events [6, 7]. There are two available SIPNI versions, desktop and web (online system), that depend on a structural context and suitable processes for their operation [6, 7].

SIPNI decentralization has been underway throughout the Brazilian territory, with expectations of advances in vaccination practices in the daily life of municipal services [8]. Nonetheless, challenges are observed regarding technology operationalization, completeness and quality of data (double entries and under-recording), and guarantee of information confidentiality and interoperability standards [9–11]. Moreover, it is slowed down by the scarcity of trained human resources, information technology deficit, and inefficiency of constant HIS updates and their integration [1, 10, 12].

Nursing professionals, who are responsible for all activities in vaccination rooms in Brazilian public services, present difficulties in the operationalization of SIPNI, which is not always easy to use. Furthermore, there are structural matters and the existence of private information systems in some municipalities that do not have compatible programming languages with SIPNI [8]. This set of issues affects the effectiveness of information systems in the production, use, and dissemination of knowledge [13].

In order to achieve that, the implementation of an HIS depends on an adequate structural context to carry the process out and obtain the expected results [14]. An adequate structure includes: availability of human resources, equipment, furniture, consumables, regulations, and personnel training. These resources ensure the performance of activities (process) that encompass acts such as software installation according to minimum requirements, training of staff, data input, processing, analysis and publication. It is expected, thus, that it result in a convenient, reliable and universal information system for the vaccination activities in daily healthcare services [1, 8]. In that sense, it is important to assess SIPNI implementation, since the evaluation results may support technology performance to optimize the workflow in vaccination rooms, by decreasing time spent with vaccination records, avoiding administration of unnecessary doses, ensuring safety whenever monitoring post-vaccination adverse effects, and keeping updated records of vaccinated patients.

Furthermore, there is currently no publicized assessment about SIPNI implementation in Brazil. Thus, the potential structural and process factors that influence system performance are unknown, as well as the growing difficulties that need to be identified and solved. Considering the complexity of HIS in Brazil, assessing its implementation is necessary for the ideal performance of a comprehensive information system and of integral healthcare, which is required to supply the demands from the National Immunization Program [8].

For those assessment needs, evaluative research [14] has been gaining notoriety, since the knowledge that is generated in this kind of investigation can contribute to the identification of problems related to organization and operation of systems, as well as support decisions aiming at their improvement and consolidation. In this study, evaluative-approach implementation analysis type 1-b was carried out, consisting of studying the relations between an intervention (SIPNI implementation) and its surrounding context to cause effects [14].

This System implementation process has involved all instances of the Brazilian Unified Health System (SUS – *Sistema Único de Saúde*) for the development of a great effort due to regional differences in the country. In the state of Minas Gerais (MG), SIPNI decentralization is at an advanced stage, but it still faces challenges regarding its operationalization and use. In view of the above, this study aims to evaluate the municipal SIPNI implementation in the state of Minas Gerais, Brazil.

## Methods

This an evaluative research focusing on the contextualized analysis of program implementation type 1-b, that consists of studying the relationships between an intervention and its context during its implementation [14]. This evaluative approach aims to determine the factors that facilitate or compromise implementation, according to the dimensions of structure (resources in use and their organization), process (performed activities) and expected results [14]. In this study, SIPNI was analyzed considering the structure and process dimensions.

A cross-section study was carried out in Primary Health Care (PHC) vaccination rooms of the 54 municipalities in the Western Region of the state of Minas Gerais. This region is located between the Central, South, and Upper Paranaíba regions. It has a vast territorial extension, with 31,543 km^2^, a medium-high Human Development Index (HDI), and a diverse economy. Regarding population size, this region consists of 58.3% small municipalities, 37.5% medium-sized municipalities and only 4.2% large municipalities, according to the classification of the Brazilian Institute of Geography and Statistics (IBGE) [15]. Regarding Family Health Strategy (FHS) coverage, 89.6% of the municipalities present FHS coverage above 80%. The region under study is composed of six health management regions, considering the territorial base of health care planning [15].

This region was chosen for study because its population has presented, in the last 10 years, the third highest population growth rate in Minas Gerais (14.93%), reaching 1,364,023 inhabitants, which corresponds to 5.8% of its total [15]. It is responsible for providing feedback on state epidemiological data, including those related to NIP.

Vaccination rooms were deemed eligible for the study sample according to the following inclusion criteria: being connected to SIPNI, employing a professional who knows how to operate the system and who is available to answer questions, besides accepting to take part in the research. All activities carried out in Brazilian vaccination rooms are under technical responsibility of the Nursing team. Nursing technicians and/or assistants, under a nurse’s supervision, perform all actions related to the vaccination room, including those regarding SIPNI [16]. Out of 307 vaccination rooms, 199 were included in the study (64.8%) and 108 (35.2%) were excluded, since they did not meet the aforementioned inclusion criteria.

Data were collected through documents (meeting minutes, reports, notifications), multidimensional questionnaire [additional file 1] filled in by a nursing professional in charge of SIPNI functions in each of the eligible rooms (*n* = 199), and direct field research observations. Data collection was carried out by the researchers in person, in a private room of each healthcare unit, after participants had been informed about the research project and signed the Free and Informed Consent Term (FICT).

The multidimensional questionnaire in use presented evaluation questions that had their content and appearance validated by Delphi Technique in a previous study [17]. This questionnaire was organized in three parts: (1) nursing professional profile characteristics; (2) structural dimension; and (3) process dimension. The questionnaire presents objective questions about the following variables: (1) sociodemographic characteristics: professional category (nurse, nursing technician or nursing assistant), professional’s age (20 to 29 years old; 30 years old or older), work experience in vaccination rooms (< 1 year; 1 to 5 years; 6 to 10 years; > 10 years), and time elapsed since graduation (< 1 year; 1 to 5 years; 6 to 10 years; > 10 years); (2) structural dimension: presence of an internet-connected computer, instruction manual, software version, IT professional for technical support, trained healthcare professional, use of communication channels to obtain system information; and (3) process dimension: regarding SIPNI Management component, there were the operation and the information analysis/publication subcomponents (immunizer records, population records, backup routine, backup storage, and monthly export of files to be sent for SIPNIS municipal coordination for the former; administered dosage report, active search for absentee patients, vaccine coverage monitoring report, use of information generated by SIPNI for inventory control of immunobiological agents, dropout rate calculation, SIPNI information disclosure for the latter), as well as the Immunized Patient Record component (immunized information record, existence of previous vaccination record e vaccination release) and the Movement of Immunobiological component (updated vaccine batch record in SIPNI, records of bottles received and used in vaccination rooms, and system input of losses of immunobiological agents).

Furthermore, regarding context variables, two of them were analyzed: (1) FHS coverage (coverage equal to or under 80%, and coverage over 80%) [18]; and (2) population size (under 10,000 inhabitants, 10,000 to 50,000 inhabitants, and over 50,000 inhabitants). This stratification was based on the references provided by the Action on Health Surveillance Qualification Program (PQAVS: *Programa de Qualificação das Ações de Vigilância em Saúde*) [19]. Analysis attempted to determine whether FHS coverage and population size influenced the Implementation Degree (ID) of municipal SIPNI. The working hypothesis was that municipalities of smaller population size could present worse ID of SIPNI, due to lacking management capacity and resources, both material and human, to work with the system. Regarding FHS coverage, there was an assumption that the higher a municipality’s coverage was, the higher ID would be observed in its SIPNI. Therefore, the working hypothesis was that PHS organization and the presence of Community Health Agents (CHAs) contribute to satisfactory SIPNI implementation and use in healthcare units.

For SIPNI ID classification, an analysis and judgment matrix was also validated by the Delphi Technique [17]. It contains questions regarding the structure and process dimensions, according to three components that are evaluated according to the logic model [17] built for System implementation assessment: SIPNI management activities, Vaccination patient records, and Movement of immunobiological. For each evaluative question a criterion, a calculation method, a parameter, an assigned value, and a cut-off point were defined to analyze whether or not the observed values complied with established standards.

Municipal SIPNI ID was defined through a score system, developed by the researchers and experts in SIPNI and HIS, using the consensus technique. This score system has different weights for each selected criterion, according to the level of importance of each one (analysis matrix). At first, observed values (Σ of the criteria scores) were determined and the implementation degree (Σ observed/Σ of maximum expected points X 100) were calculated for each component. After that, total ID was calculated by adding up all components.

Scores, as obtained from the addition of criterion points in each dimension, were turned into percentages in reference to the maximum possible score. ID categories were defined from these percentages into four groups: Adequate implementation (80 to 100%); partially adequate implementation (60 to 79.9%); Inadequate implementation (40 to 59.9%); and Critical implementation (under 40%). The closer to 100% the ID, the more appropriate is the SIPNI implementation.

Analysis of structure and process dimensions according to their components and subcomponents, separately, was performed through adequation of calculated average values in relation to the maximum points assigned to each criterion. The measure of position in use for the variables (age, work experience in vaccination rooms and time elapsed since a professional’s graduation) was the median, as adequate for the analysis of asymmetric distributions.

With the purpose of describing and reaching an overview of how SIPNI implementation has been advancing on the regional territory, the average value was calculated for the SIPNI ID of each municipality as a group, by each of the health regions. In order to assess the relationship between SIPNI ID and external context variables (FSH coverage and population size), Pearson’s Chi-Square Test was used with a significance level of 95%.

Data were processed using Epidata (version 3.1, Epidata Association, Odense, Denmark) and analyzed through the Statistical Package for Social Sciences Software (SPSS) version 21.0. In order to ensure higher quality, double typing was performed and, later, comparison and validation of this double typing was made with the Epi info software (version 3.5.1. Epi info™, Centers for Disease Control and Prevention, United States).

This study was approved by the Research Involving Human Beings Ethics Committee of *Universidade Federal de São João del-Rei* (REC/UFSJ) under Opinion 2,000,305 and CAAE (*Certificado de Apresentação para Apreciação Ética* - Certificate of Presentation for Ethical Consideration) 65,656,017,60,000,5545.

## Results

Out of 307 eligible vaccination rooms, 199 (64.8%) presented SIPNI implemented in them, 94 (30.6%) used a different information system and 14 (4.6%) used no IIS. The 199 rooms with implemented SIPNI are spread throughout 48 municipalities in the studied Region.

Most nursing professionals who took part in the study (*n* = 199) were female (189; 95%), aged between 23 and 63 years (155; 77.9%) on a 36-year-old median. Nearly all of them worked with FHS (192, 96.5%) and the median value of working experience in vaccination rooms is of 6 years. Among 108 nurses (54.3%), 70 of them held the title of specialists (64.8%) and only one of them has a master’s degree (0.9%). Time elapsed since a professionals’ graduation varied from < 1 year to 35 years, presenting a median value of 10 years.

**Fig. 1:**
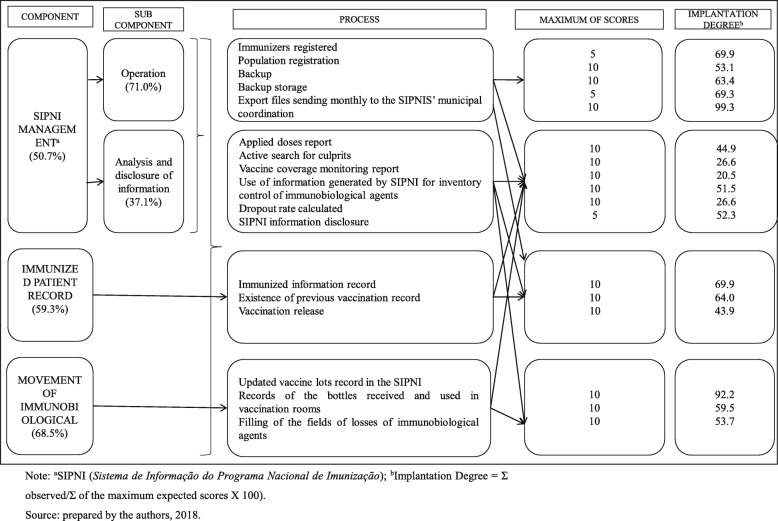
ID distribution in the assessment of criteria of the process dimension, according to SIPNI components, in vaccination rooms (*n* = 199) of the Western Region of Minas Gerais, 2017

Upon analysis of the 199 SIPNI-implemented vaccination rooms, “structure” dimension had a better evaluation than “process” dimension, with respective IDs of 70.9 and 59.5%, as presented on Table 1 and Fig. 1.

Regarding structure, out of the seven criteria under analysis, the only one rated adequate was the presence of a computer in the vaccination room (Table 1). All identified computers in vaccination rooms hold minimal technological requirements (Processor, Hard Disk, Floppy disk, Optical unit, Memory) to provide adequate SIPNI operation.

Use of communication channels to solve doubts about SIPNI was the worst evaluated criterion. Although there are available communication channels, such as interactive technologies, SIPNI DATASUS chat site and SIPNI videotapes on YouTube, they are seldom used.

Regarding the structure dimension, the criteria deemed “partially adequate” include the existence of a SIPNI manual; internet access, although 34.7% of healthcare units reported connection instability; predominance of the desktop version; existence of a computer science professional for technical support; and presence of a trained professional, despite the fact that, out of the 32.1% provided training courses, 25.1% were only carried out in the presence of new versions of System upgrade (Table 1). It is noteworthy that in 100% of vaccination rooms the use of paper forms to record vaccination activities is still maintained, even in vaccination rooms that use SIPNI web, thus pointing out failures in its operation.

**Table 1:** ID distribution in the criteria assessment of SIPNI structure dimension, in vaccination rooms (*n* = 199) of the West Region of Minas Gerais, 2017

Criteria	Maximum points	Implantation Degree^**b**^
Existence of a computer in the vaccination room	10	89.3
Existence of SIPNI^a^ manual (online or printed)	5	74.5
Existence of professional for technical support (computer science)	5	72.1
Use of communication channels^c^	5	27.9
Trained Professional	10	70.5
Internet connection	10	67.3
SIPNI desktop version	10	63.1
Total	**55**	**70.9**

^a^SIPNI (*Sistema de Informação do Programa Nacional de Imunização*); ^b^Implantation Degree = Σ observed/ Σ of the expected maximum points X 100); ^c^The communication channels are the means used by the professionals who operate the information system in order to obtain information, training and support to adequately navigate a system, SIPNI in case. E.g.: video classes, instant messaging, telehealth, video conferences, chats, etc.

In the process dimension, Movement of Immunobiological was the best-rated component (ID = 68.5%), followed by immunized patient records (ID = 59.3%). SIPNI management was found inadequate (ID = 50.7%) and its subcomponents – operationalization and analysis, and information dissemination – were deemed partially adequate and in critical implementation status, respectively (ID = 71.0%, ID = 37.1%) (Fig. 1).

Results point towards the dependence relation between assessed components. “SIPNI management” component was influenced by “vaccination patient records” and “Movement of Immunobiological” components. For instance, if there is no “updated records of vaccine batches in SIPNI”, it is impossible to issue “useful reports for immunization activity monitoring” and “record vaccination patient data”. Another example refers to the relation of subcomponent “information analysis and publication” with subcomponent “SIPNI operationalization” which, faced with gaps on “register of population under care”, highly impairs “report generation” and “vaccination dropout rate calculation”.

In the process dimension, the only SIPNI activities that were adequately carried out were the update of vaccine batch records in SIPNI and monthly export of files for municipal coordination. Other activities – such as recording bottle input/output in vaccination rooms, filling in forms about immunobiological material losses, disclosing information produced by SIPNI, registering population under care, using information generated by SIPNI for inventory control of immunobiological materials, issuing administered dosage reports, and controlling vaccine schedules – were insufficient and therefore deemed inadequate. Criteria evaluated as in critical condition correspond to issuing reports to monitor the active search for absentees, monitoring the dropout rate, and issuing reports to monitor vaccine coverage.

The other evaluated criteria were rated as partially adequate. Attention should be paid to the criteria “existence of prior records of vaccines” and “registration of immunized patients’ information”, which, although deemed partially adequate, are still insufficient to ensure SIPNI performance.

The 199 SIPNI-implemented vaccination rooms are spread throughout 48 municipalities. It was noticed that only 2.1% of those municipalities presented an adequately implemented SIPNI; 64.6% presented partially adequate implementation; 22.9% had inadequate implementation; and 10.4% showed critical implementation status. It was observed that few vaccination rooms operated out of SIPNI web version (online) (59; 29.6%); most used the desktop version (140; 70.4%).

Upon analysis of the 48 municipalities divided into six health management regions, it was observed that in five of those regions SIPNI implementation was deemed partially adequate, and one region presented inadequate implementation (Table 2).

**Table 2:** Classification of SIPNI ID, according to the health regions of the Western Region of Minas Gerais, 2017

Health Region (***n*** = 199)^**a**^	Implantation Degree^**b**^	Classification
Region A (*n* = 27)	64.2%	Partially adequate
Region B (*n* = 42)	65.9%	Partially adequate
Region C (*n* = 47)	63.1%	Partially adequate
Region D (*n* = 35)	61.1%	Partially adequate
Region E (*n* = 30)	70.2%	Partially adequate
Region F (*n* = 18)	48.4%	Inadequate

^a^vaccination rooms; ^b^Implantation Degree = (Σ observed/ Σ of the maximum points expected X 100)

In the bivariate analysis, it was observed that the context variables, FHS coverage and population size, did not have a statistically significant association with municipal SIPNI ID (*p* = 0.606; *p* = 0.781). Therefore, they did not influence SIPNI implementation in the analyzed municipalities (Table 3).

**Table 3:** Association between external context (Family Health Strategy coverage and population size) and SIPNI ID in municipalities (*n* = 48) of the Western Region of Minas Gerais, 2017

External Context			Implantation Degree^**a**^						
	Adequate		Partially adequate		Inadequate		Critical		***P*** value^**b**^
**FHS Coverage**	N	%	N	%	N	%	n	%	
Up to 80%	0	0.0	2	40.0	2	40.0	1	20.0	0.606
> 80%	1	2.3	29	67.4	9	20.9	4	9.3	
**Population Size**									
< 10 thousand inhabitants	0	0.0	19	67.9	6	21.4	3	10.7	0.781
Between 10 and 50 thousand inhabitants	1	5.6	10	55.6	5	27.8	2	11.1	
50 thousand inhabitants and more	0	0.0	2	100.0	0	0.0	0	0.0	

^a^Implantation Degree = Σ observed/Σ of the expected maximum points X 100); ^b^Pearson’s Chi-Square Test with a 95% significance level

## Discussion

Municipal SIPNI in the Western Region of MG is not adequately implemented and that is mainly due to the way activities are conducted in health service units, signaling problems in the use of technology by professionals who work in vaccination rooms. The results of this study point out that the partial performance of the System is independent of population size of municipalities and of FHS coverage. On the other hand, it emphasizes the dependency relation between activities carried out on the evaluated components (SIPNI management, immunized patient record and Movement of Immunobiological) with structural conditions of health services.

Lack of standardization of local information management, insufficient access to the Internet, inadequate use of communication channels to solve doubts about SIPNI operation, lack of a professional qualification policy and a permanent education program are some of the structural impediments to good SIPNI performance, corroborating the scientific literature that evaluated the HIS [1–3, 12, 13]. Other studies emphasize incompleteness of data, fragmentation in health care procedures, costs of technology (software), financial issues, power outages, slow internet connection, lack of knowledge and training by professionals who operate the systems, time required to input data, use of paper-based forms to record data, and the inability to generate reports and to use system functionalities as the main obstacles to the use and implementation of HIS in health services, especially in PHC [20–25].

It is known, however, that despite some difficulties in structural factors of SIPNI consolidation, this reality is not exclusive to the state of Minas Gerais in Brazil. Currently, SIPNI web version is also under implementation in some Brazilian cities, which face important challenges, including the requirement of internet access of good quality [8].

In the “process” dimension, the best-evaluated activity was the monthly exports of vaccination-related files to municipal coordination, which are requisite so health units can receive financial backing. This reality was also observed in a study that was carried out in Brazil [26] that reports on IIS users seeing data only as necessary steps to comply with a policy/program and reach goals linked to financial support.

In this context, the results observed in this study indicate that activities related to SIPNI management, such as analysis and dissemination of information, are not completely carried out. Essential reports for planning immunization actions are not yet issued to monitor vaccination coverage, active search for absentees, dropout rates and administered doses. The study assumes that most municipalities that have low vaccine coverage and/or high risk of immune-preventable diseases have as their main cause the inappropriate IIS implementation. This is probably due to the double input of administered doses, incompleteness of data, lack of individual immunized patient records, and failure to perform the active search for absentees [27].

Most of the time, computer systems are seen as mandatory bureaucratic tools, whose function is to collect data from the local level and export them to hierarchically superior levels [10, 28]. Nonetheless, it is necessary to guide professionals into understanding the importance of collected data and into discussing them in daily life, thinking about how one can use this information to take care of collective health [11, 29]. Therefore, there should be training and skill-building not only for the operationalization of HIS, but also for the use of generated information, making it a powerful management tool [1, 26, 30–32].

SIPNI is a technological innovation that has multiple advantages, besides allowing interoperability with other HISs by reading and inputting records generated by them [6]. Interoperability is the HIS capability of working together independently of organizational limits, sharing information in and effective and efficient way, aiming at progressing to the effective provision of healthcare to individuals and communities [2, 33]. Other HISs of countries such as Canada, England and the United States of America (USA) are also considered technological innovations that hold many functionalities, among which there is data protection through the use of several servers, what makes databases reliable and interoperability still rudimentary [5, 34].

HIS in the USA performs vaccination coverage analysis both from area and age of patients under coverage, and from individual vaccination records, similarly to the Brazilian HIS under study (SIPNI). Moreover, that information system in the USA has some additional functions in relation to SIPNI, which allow patients to follow their vaccination status through reminders and notifications about the next vaccine doses to be administered, showing the list of vaccines provided by the State and supporting clinical decisions for vaccinations through algorithms of vaccine prediction, based on recommendations from the Advisory Committee of Immunization Practices (ACIP) [5].

In that sense, it is necessary to develop information systems, such as the aforementioned ones, that have interoperability as a basic requirement and an interface that may answer to the professionals’ real necessities. It is also pivotal that, in healthcare units, there be good internet connectivity to the Internet so data input time becomes shorter and professionals may dedicate more to providing care [35]. The availability of internet connectivity in Brazil is still a challenge in some areas due to its diversity and land size [36].

In that sense, the federal government has started “National Broadband Program”, aiming at making broadband internet connection available for all primary healthcare units, resulting in greater speed and care quality, ensuring safety on HIS data transmission processes, as well as providing convenience in real-time information access [37].

As limitations of the study, we highlight the fact that a single quantitative approach was used to evaluate SIPNI implementation. Subjective aspects related to the intentionality of manipulating the system may interfere with the process and, once explored further, may reveal other responses and tendencies. New qualitative-based research should be performed in order to analyze the intrinsic elements related to the professional who uses this tool.

As a strength, the methodological approach generates an overview of the study object, which is SIPNI implementation and can serve as a guiding axis for new research that evaluates HIS performance. In addition, the results of this study can support the sustainability of SIPNI as a necessary innovation technology for the optimization of activities in vaccination rooms, such as reducing the time spent with vaccination records, administering unnecessary doses, post-vaccination adverse event monitoring, and updated immunized patient records. In the medium and long term, the study is expected to foster discussions to promote safety culture in vaccination rooms.

## Conclusions

SIPNI is a tool that potentializes the performance of healthcare managers and providers in the dynamic risk assessment of infection outbursts or epidemics, based on the records of administered immunological materials and on the quantity of vaccinated people, grouped according to age, timeframe and geographic location. However, the results have shown that this information system for immunization is not adequately implemented in Brazil, according to the studied scenario.

It is also important to highlight that the study raised the interest of professionals who work in vaccination rooms about SIPNI functionalities. Consequently, interventions about its use and potentialities were carried out during the research time, favoring technological spread. Therefore, challenges remain to be overcome, such as the implementation of the final web version, internet connectivity, and training towards the use of information generated by the technology. Nevertheless, perspectives regarding system use are positive, since it is a technological innovation with useful functionalities in the vaccination room, besides interoperability with other systems, what leads to the belief that SIPNI shall connect to other systems included in the e-SUS strategy.

## Supplementary information

**Additional file 1.** Immunization Information System Assessment Form. It is a multidimensional questionnaire in use presented evaluation questions that had their content and appearance validated by Delphi Technique in a previous study. This questionnaire was organized in three parts: (1) nursing professional profile characteristics; (2) structural dimension; and (3) process dimension.

## Data Availability

Data sets used and/or analyzed during the current study may be made available by the corresponding author upon reasonable request.

## Abbreviations

HIS: Health Information System
IIS: Immunization Information System
USA: United States of America
NIP: National Immunization Program
DATASUS: *Departamento de Informática do Sistema Único de Saúde*
SIPNI: *Sistema de Informação do Programa Nacional de Imunização.*
SUS: *Sistema Único de Saúde.*
MG: Minas Gerais.
PHC: Primary Health Care.
HDI: Human Development Index.
IBGE: *Instituto Brasileiro de Geografia e Estatística.*
FHS: Family Health Strategy.
ID: Implementation Degree.
FICT: Free and Informed Consent Term.
PQAVS: *Programa de Qualificação das Ações de Vigilância em Saúde.*
CHA: Community Health Agent.
SPSS: *Statistical Package for Social Sciences Software.*
UFSJ: *Universidade Federal de São João del-Rei.*
