# Supplementary material for: National Immunization Program Information System: implementation context assessment
Source: BMC Health Serv Res. 2020 Apr 21;20:333. doi: 10.1186/s12913-020-05175-9 (PMC7171780; doi:10.1186/s12913-020-05175-9)
Supplement: Supplementary file 1 — Additional file 1. Immunization Information System Assessment Form. It is a multidimensional questionnaire in use presented evaluation questions that had their content and appearance validated by Delphi Technique in a previous study. This questionnaire was organized in three parts: (1) nursing professional profile characteristics; (2) structural dimension; and (3) process dimension. [file 12913_2020_5175_MOESM1_ESM.docx]

| **IMMUNIZATION INFORMATION SYSTEM ASSESSMENT FORM (IISAF)** |
| --- |
| **Socio-demographic Characterization** |
| 1. Municipality: |
| 2. Health Unit: |
| 3. Age: |
| 4. Sex:  Female  Male |
| 5. Graduation Year (assistance course, technical course or undergraduate): |
| 6. Professional Category:  Nurse  Nursing Technician  Nursing Assistant  Community Health Agent  Others: |
| 7. Place of Work  Family Health Strategy (ESF)  Health Center  Community Health Agents Strategy (EACS)  Vaccine conservation cold chain Center  Others: |
| 8. Highest Degree Completed:  Elementary School (Nursing Assistant)  Technical High School (Nursing Technician)  Undergraduate Degree  Specialization  Master`s Degree  Doctoral Degree  Postdoctoral Degree |
| \| * In case the amount of years is less than one, you should specify the number of months (add “months” after the number) \| \| --- \|   9. How many years have you been working in the vaccination room? |
| 10. In a scale of 0 to 10, how would you grade your knowledge about SIPNI: |
| 11. What is the information system used in vaccination rooms to register information:  SIPNI  Each municipality uses its own information system |
| 12. Number of practitioners working at the vaccination room: |
| 13. Number of vaccinators (individuals who administer vaccines) in the health unit: |
| 14. Number of vaccinators (individuals who administer vaccines) registered in SIPNI: |
| 15. Number of practitioners registered in SIPNI who do not administer vaccines: |
| 16. Is the SIPNI operator someone from the health unit itself?  Yes  No |
| 17. SIPNI implementation date: |
| 18. Is the information registered in paper as well as in the information system?  Yes  No |

| **Structural Dimension** | |
| --- | --- |
| *Component* | Evaluation questions |
| SIPNI Management  Immunized Patient Records  Movement of Immunobiological | 19. Is a computer with SIPNI being used in the vaccination room?  Yes  No |
|  | 20. Is the SIPNI manual available for consultation by practitioners (online or in hard copy)?  Yes  No |
|  | 21. Is there a technical professional offering SIPNI related support when needed?  Yes  No |
|  | 22. Have you ever used some kind of communication channel to ask for SIPNI related support?  Yes  No |
|  | 23. Were you trained to operationalize SIPNI?  Yes, I was trained and I feel ready.  Yes, I was trained but I do not feel ready.  No, I was not trained but I feel ready.  No, I was not trained and I do not feel ready. |
|  | 24. Does the vaccination room have access to the internet?  Yes, with stable connection  Yes, but with unstable connection  No |
|  | \| * This question applies only to the vaccination rooms that have SIPNI supplied by the Health Ministry and not to the ones with their own system. The vaccination rooms with their own system should mark the last option (shall not apply). \| \| --- \|   25. What SIPNI version is being used?  Web Version (online)  Desktop Version  Shall not apply (use of our own information system) |

| **Process Dimension** | | |
| --- | --- | --- |
| *Component* | Subcomponents | Evaluation questions |
| SIPNI management | Operation | 26. Are all the practitioners working in the vaccination room registered in SIPNI as vaccinators?  Yes  No |
|  |  | 27. Are the people in the area of influence of this health unit registered in SIPNI?  Yes, everyone.  No, only the ones receiving vaccines.  We do not register. |
|  |  | 28. Is a backup of SIPNI data done at the end of the workday?  Yes, daily.  Yes, but not daily.  Never  Shall not apply (SIPNI Web version)  Shall not apply (use of our own information system) |
|  |  | 29. Where is the safety copy of the SIPNI data stored?  Outside the computer (pen drive, cloud, CD, DVD, etc.).  Another folder in the same computer.  SIPNI standard folder.  We do not make safety copy.  Shall not apply (SIPNI Web version)  Shall not apply (use of our own information system) |
|  |  | 30. Do you send the export files monthly to SIPNI municipal coordination?  Yes  No  Shall not apply (SIPNI Web version)  Shall not apply (use of our own information system) |
|  | Information analysis/publication | 31. Is a report generated to monitor the applied doses?  Monthly  Quarterly  Six-monthly  Annually |
|  |  | 32. Is a report generated to monitor the listing of people with late vaccination record cards (defaulting)?  Monthly  Quarterly  Six-monthly  Annually |
|  |  | 33. Is a report generated to monitor the vaccination coverage?  Monthly  Quarterly  Six-monthly  Annually |
|  |  | 34. Is the information generated by SIPNI used to control the immunobiological inventory?  Yes  No |
|  |  | 35. Is the information generated by SIPNI used to calculate the abandonment rates?  Yes  No  Sometimes |
|  |  | 36. Is the consolidated information produced in SIPNI disclosed to the public?  Yes  No |
| Immunized Patient Records |  | 37. Is the optional record of the vaccinated in the vaccinated record window done?  Yes  No |
|  |  | 38. Is the record of the previously administered vaccine (previous record) done in SIPNI?  Yes  No  Sometimes |
|  |  | 39. When the administered vaccine does not have automatic scheduling in SIPNI, do you make the scheduling manually in the system?  Yes  No  Sometimes |
| Movement of Immunobiological |  | 40. Is the register of vaccine batches in SIPNI updated?  Yes  No |
|  |  | 41. Is the number of vaccine vials received and used in the vaccination room recorded in SIPNI?  Yes  No |
|  |  | 42. Do you fill the immunobiological loss fields in the proper module in SIPNI?  Yes  No |
